# Temporal dynamics of the hummingbird-plant interaction network of a dry forest in Chamela, Mexico: a 30-year follow-up after two hurricanes

**DOI:** 10.7717/peerj.8338

**Published:** 2020-01-09

**Authors:** Sergio Díaz Infante, Carlos Lara, Maria del Coro Arizmendi

**Affiliations:** 1 Laboratorio de Ecología, UBIPRO, Facultad de Estudios Superiores Iztacala, Universidad Nacional Autónoma de México, Tlalnepantla de Baz, Estado de México, Mexico; 2 Centro de Investigación en Ciencias Biológicas, Universidad Autónoma de Tlaxcala, San Felipe Ixtacuixtla, Tlaxcala, Mexico

**Keywords:** Interaction networks, Hummingbirds, Temporal dynamics, Hurricanes, Mexico, Conservation, Hummingbird–plant interactions, Bird-pollination

## Abstract

**Background:**

Interactions among species are a driving force of community structure. The species composition of animal-plant interaction networks can be highly dynamic on a temporal scale, even though the general network structure is usually not altered. However, few studies have examined how interaction networks change over long periods of time, particularly after extreme natural events. We analyzed herein the structure of the hummingbird-plant interaction network in a dry forest of Chamela, Mexico, comparing the structure in 1985–1986 with that in 2016–2017 following the passage of two hurricanes (category 2 Jova in 2011 and category 4 Patricia in 2015).

**Methods:**

The fieldwork was carried out in the Chamela-Cuixmala Biosphere Reserve in Jalisco, Mexico. In the last 30 years, three severe drought events and two hurricanes have affected this region. Previously, from 1985–1986, hummingbird-plant interactions were recorded monthly for one year in the study area. Then, from 2016–2017, we replicated the sampling in the same localities. We compared the network parameters describing the plant-hummingbird interactions of each period using adjacency matrices.

**Results:**

We found differences in the number and identity of interacting species, especially plants. The plant species missing in 2016–2017 were either the least connected in the original network (1985–1986) or belonged to groups such as cacti, epiphytes, or trees. The new plant species incorporated in the 2016–2017 network were herbs, vines, and shrubs, or other species barely connected. These changes in the composition are consistent with reports on vegetation damage after strong hurricanes at other study sites. Conversely, all hummingbird species remained in the network, with the exception of *Heliomaster constantii*, which was primarily connected to a plant species absent in the 2016–2017 network. Migratory and habitat generalist species (i.e., *Archilochus* spp*.*) showed higher abundances following the disturbance events.

**Conclusions:**

Most of the parameters describing the hummingbird-plant network structure remained unchanged after 30 years, with the exception of an increase in plant robustness and hummingbird niche overlap. However, the network’s generalist core was affected by the loss of some species. Also, core plant species such as *Ipomoea bracteata*, *Combretum farinosum*, and *Justicia candicans* were found to be important for maintaining the hummingbird-plant interaction network. The temporal perspective of this study provides unique insights into the conservation of plant-hummingbird networks across time and extreme natural events.

## Introduction

Drought and extreme winds are the main natural disturbances in Neotropical dry forests (Durán et al., 2002). Natural factors such as these have been found to affect plant–pollinator interactions. It is likely that natural disturbances such as drought and hurricanes will only continue to intensify in the near future as a result of climate change (Allen et al., 2010; Knutson et al., 2015). Therefore, it is important to investigate the effects of these natural disturbances on biodiversity and ecosystem functioning, including plant-animal interactions.

Interactions among species are the driving force behind the biodiversity of the Earth (Bascompte, 2009) as well as the structure of its communities (Berlow et al., 2009).These interactions can be represented as networks, enabling the topology (or patterns) of the relationships among species to be characterized. Accordingly, these networks provide a simple and powerful framework for understanding species interactions (Poisot, Stouffer & Gravel, 2015). Additionally, network theory has informed several approaches for statistically quantifying and comparing patterns across communities (Bascompte & Jordano, 2007). These approaches are essential for understanding the ecological, evolutionary, and co-evolutionary dynamics of a network that cannot simply be deduced from pairs of interacting species (Bascompte, 2009).

At a basic level, network structure reflects the organization of species interactions according to the frequency of interactions per node (Proulx, Promislow & Phillips, 2005; Bascompte, 2009). Overall, species interactions tend to be highly heterogeneous: Few species have many interactions, and many species have only a few interactions. In particular, plant–pollinator networks have several common features (Bascompte et al., 2003; Lewinsohn et al., 2006; Guimarães et al., 2007) as well as specific ecological characteristics and structure (Bascompte et al., 2003; Lewinsohn et al., 2006; Guimarães et al., 2007; Morales & Vázquez, 2008; Vázquez et al., 2009). For example, plant–pollinator networks tend to be nested: Generalist species mainly interact with other generalists, forming a core, and specialists also mainly interact with generalists and rarely with other specialists (Bascompte et al., 2003). These patterns of interaction can be explained by ecological processes and evolutionary history (Poisot, Stouffer & Gravel, 2015; Díaz-Castelazo et al., 2010).

However, there is a scarcity of research on how networks change over long periods of time, especially after extreme natural events (Dupont et al., 2009; Díaz-Castelazo et al., 2010). The few studies examining temporal changes in species interaction networks have reported that these networks retain basic topological properties even when plant–pollinator interactions vary over time (Alarcón, Waser & Ollerton, 2008). Notably, species interaction networks that show high species turnover and low variation in their structural parameters (Petanidou et al., 2008; Dupont et al., 2009; Luviano et al., 2018) may be less sensitive to disturbance effects as a result of climatic changes and hurricanes.

Natural events have several wide-ranging effects on natural vegetation that may affect animal-plant interaction networks. In dry forests, most plant species are adapted to drought (Meir & Pennington, 2011). However, extreme winds during hurricanes can result in vegetation damage or fallen trees, which create forest gaps (Askins & Ewert, 1991). Also, vegetation may be severely defoliated, and most flowers and fruits may be dropped (Lynch, 1991; Waide, 1991). Such changes to the original canopy structure may further affect the production of flowers, the primary food resource for pollinators (Askins & Ewert, 1991). Following natural events, greater (major) defoliation has been observed in the superior canopy strata compared to the lower canopy strata and understory, where re-growth tends to be faster (Wunderle Jr, Lodge & Waide, 1992; Brokaw & Walker, 1991).

Aside from vegetation damage, a hurricane can kill birds as a result of wind and rain exposure (Wiley & Wunderle, 1993). Surviving birds can be debilitated and subjected to additional mortality or predation (Lugo, 2008). After Hurricanes Gilbert and Hugo, several studies reported that resident nectarivorous birds were among the most affected (Askins & Ewert, 1991; Lynch, 1991; Waide, 1991; Wunderle Jr, Lodge & Waide, 1992). Nonetheless, the subsequent recovery of their populations to previous numbers suggests that reductions in their populations were due to regional movements as individuals searched for food rather than mortality (Waide, 1991). After Hurricane Gilbert in Jamaica Wunderle Jr, Lodge & Waide (1992) found that nectarivorous species diminished their numbers in the affected areas yet increased their numbers in nearby, less affected areas.

With respect to hurricanes, most ecological studies have focused on their impacts on vegetation (Baldwin et al., 1995; Lomascolo & Aide, 2001), animal communities (Covich et al., 1991; Lugo, 2008), or both (Ferguson et al., 1995; Pascarella, 1998; Rathcke, 2001; Angulo-Sandoval et al., 2004; Horvitz, Tuljapurkar & Pascarella, 2005) although, as previously mentioned, few have documented the effects on species interaction networks (Sánchez-Galván, Díaz-Castelazo & Rico-Gray, 2012; Luviano et al., 2018). In one case, Sánchez-Galván, Díaz-Castelazo & Rico-Gray (2012) studied the effect of Hurricane Karl on a plant-ant interaction network in Veracruz, Mexico. These authors found that most changes occurred in the presence and proportions of interacting species. Also, Luviano et al. (2018) documented the impact of Hurricane Jova on plant-herbivore interaction networks along a successional chronosequence in a dry forest of the Chamela region in Jalisco, Mexico. These authors found that some network descriptors were altered; however, the overall network topology associated with forest succession remained unaltered.

We studied herein the hummingbird-plant interactions in a dry forest of Chamela, Mexico. Our aim was to assess temporal changes in (1) the number and identity of interacting species and (2) the topology of the interaction network over a 30-year period during which several disturbances occurred. Considering the results of previous studies (Alarcón, Waser & Ollerton, 2008; Petanidou et al., 2008; Dupont et al., 2009; Díaz-Castelazo et al., 2010; Dupont et al., 2009; Luviano et al., 2018), we expected that the hummingbird-plant interaction network in the dry forest of the Chamela Biological Station would maintain most hummingbird species over time and that greater changes would be observed in plant species (including the generalist core), mainly due to changes in vegetation cover caused by the passage of Hurricanes Jova and Patricia. As a consequence, we also expected changes in the number and identity of some interactions (i.e., more connections between hummingbirds and lower-strata plants such as shrubs, vines, and herbs and fewer connections between hummingbirds and higher-strata plants such as large trees),yet we expected the general network structure to remain unaltered (i.e., that most network parameters would remain unchanged).

## Materials and Methods

### Study site

Fieldwork was carried out at the Chamela-Cuixmala Biosphere Reserve (19°22′–19°39′N, 104°56′–105°10′W), which has an extension of 13,142 ha. The reserve is located about 2 km from the Pacific coast, and its elevation ranges from 35–120 m.a.s.l. Most rainfall occurs between June and October. The mean annual precipitation is 745 mm but is variable from year to year, ranging from 366 to 1,329 mm (Bullock, 1986). The dominant vegetation is tropical dry forest (TDF), which is mainly located on hillsides. This vegetation has a dense understory and trees that remain leafless for around 5 months during the dry season (Lott, Bullock & Solis-Magallanes, 1987). Semi-deciduous tropical forest with taller trees (with an average tree height of 15 m and some trees exceeding 25 m) is also present along river basins and valleys (Lott, Bullock & Solis-Magallanes, 1987; Rzedowski, 2006). Overall, the Chamela-Cuixmala region is considered to be a highly biodiverse region, with 1,146 plant species (average height of 6 m) and a large number of endemic species (Lott, Bullock & Solis-Magallanes, 1987; Banda et al., 2016). The study site has been preserved from human disturbance; however, the forest has experienced natural disturbances such as droughts and hurricanes, although not fires (Maass et al., 2017).

In the last 33 years, there have been three severe droughts (1985–1987, 1990–1991, and 2000–2005, with a mean annual rainfall of 484, 550, and 554 mm, respectively). Even so, TDF plants are generally considered to be well adapted to high rainfall variability (Maass et al., 2017). In addition, two hurricanes have hit the area: category 2 Hurricane Jova in 2011 (Lujano & Hernández, 2011) and category 4 Hurricane Patricia in 2015 (Kimberlain, Blake & Cangialosi, 2016). The latter hurricane is the largest and strongest hurricane registered to date (Foltz & Balaguru, 2016).

### Interaction records

During one year spanning 1985 to 1986, hummingbird visits to plants were recorded monthly along permanent transects (2-km long and 100-m wide) following Emlen’s method (Emlen, 1971). These two transects were located inside the biological station, one mainly in deciduous forest (El Tejón) and the second (Eje Central) almost parallel to the dry bed of an arroyo, intersecting several patches of semi-deciduous forest. A third transect was established in a disturbed area outside the station but was excluded from the present analysis with the aim of only considering interaction records from inside the protected area.

In 2016 (May–December) and 2017 (January–April), five years after Hurricane Jova and seven months after Hurricane Patricia, we established 20 circular plots with a 25-m radius at a distance of 200 m from one another (based on their central points) (Hutto, Pletschet & Hendricks, 1986; Ralph et al., 1996) along the same transects (El Tejón and Eje Central) used in the 1985–1986 survey. At monthly intervals, point counts were performed in each plot for 10 min to record the number and species of hummingbirds feeding on flowering plants. Censuses began around 7:30 a.m., starting at a different randomly chosen point and moving in a different direction each day to avoid order effects (Ralph, Droege & Sauer, 1995). Binoculars (10 × 40 mm) and a field guide were used to identify hummingbirds (Arizmendi & Berlanga, 2014), and plant specimens were identified at the Chamela Biological Station Herbarium.

Both census methods (transects and point counts) are based on bird detection probabilities (i.e., the detection probability is higher at lower distances and vice versa), thereby allowing bird density to be estimated (Ralph, Droege & Sauer, 1995). The results from both methods are considered to be similar (Ralph et al., 1996) according to several comparative studies (e.g., Anderson & Ohmart, 1981; Edwards, Dorsey & Crawford, 1981; Verner & Ritter, 1985; Posadas-Leal et al., 2011).

Although the transect method is more appropriate in open habitats where walking is easier compared to more rugged terrains, the point count method is currently the most used method and, therefore, has been adopted as the standard method in bird monitoring (Ralph, Droege & Sauer, 1995). Recent studies (e.g., Falcão, Dáttilo & Rico-Gray, 2016) comparing interaction networks mention that the main factor affecting accuracy (consistency in the results of these methods) may be related to survey effort (duration and frequency). In our case, both surveys were performed monthly for one year along the same transects (Tejón and Eje Central) for the same amount of time (for 6 to 7 h starting in the morning over 2 days).

### Interaction networks

Pooling together the monthly hummingbird-plant interaction data from each study (1985–1986 and 2016–2017), we built two adjacency matrices, where *aij* = number of interactions between an individual plant species (*i*) and an individual hummingbird species (*j*) (i.e., number of observed visits; Dormann, Gruber & Fründ, 2008) regardless of the quantity of flowers visited. A zero value indicates no interaction (Bascompte et al., 2003). From these matrices, we built two bipartite networks of plant-hummingbird interactions using the “Bipartite” package in R software (R Development Core Team, 2017; Dormann, Gruber & Fründ, 2008). To evaluate temporal changes in the identity of species important for the network structure, we classified plant and hummingbird species as either core or periphery species using the following formula: *Gc* = (*k*_*i*_–*k*_*mean*_)/*σ*_*k*_, where *k*_*i*_ = mean number of links for a given plant/hummingbird species, *k*_*mean*_ = mean number of links for all plant/hummingbird species in the network, and *σ*_*k*_ = the standard deviation from the mean number of links for all plant/hummingbird species. Species with a *Gc* value >1 have a larger number of interactions in relation to other species of the same trophic level and are therefore considered to form part of the generalist core. Meanwhile, species with a *Gc* value <1 have a lower number of interactions in relation to other species of the same trophic level and are therefore considered to be periphery species (Dáttilo, Guimarães & Izzo, 2013).

To evaluate how the topological properties of the plant-hummingbird mutualistic networks vary over time, we calculated and compared the following metrics: connectance, robustness, network specialization, niche overlap, and nestedness. We chose these metrics because their results can provide an overview of community organization and be compared with previously published studies. Also, whole network-level metrics such as nestedness and connectance can be related to emergent properties such as network stability. Specifically, connectance (1), which is defined as the proportion of realized links out of all possible links, was measured to determine whether one of the networks was more connected than the other (Winemiller, 1989; Dunne, Williams & Martinez, 2002). Robustness (2) was measured to describe the tolerance of both networks to the extinction of their component species as a result of, for example, natural disturbance events. It represents the fraction of species that survives after one species is removed and is obtained from calculating the area under the extinction curve according to Memmott, Waser & Price (2004). In this case, we considered a random deletion of species according to their abundances, with the least abundant going extinct first (Dunne, Williams & Martinez, 2002; Memmott, Waser & Price, 2004). A robustness value (R) = 1 is consistent with a very robust system where most plant species survive even if a large fraction of animal species are eliminated; conversely, *R* = 0 is consistent with a fragile system where most plants lose their interactions and go extinct even if only a small fraction of animal species are eliminated (Burgos et al., 2007). Network specialization H_2_’ (3) characterizes the degree of resource partitioning between two parties in the entire network and is useful for comparisons across different interaction networks. It is based on the deviation of each species’s realized number of interactions from the expected total number of interactions. This latter value ranges from 0 (no specialization) to 1 (complete specialization given total interactions). Notably, this index is robust to the number of interacting species and to changes in sampling intensity (Blüthgen, Menzel & Blüthgen, 2006). Niche overlap (4) is the mean similarity in interaction patterns at the species level (calculated by default as Horn’s index) (Horn, 1966). Values near 0 indicate no common use of niches, whereas a value of 1 indicates perfect niche overlap (Krebs, 1989). Even though specialization (H_2_’) and niche overlap may likely give qualitatively similar results (Dormann, Gruber & Fründ, 2008), they do so at different levels: H_2_’ at the network level and niche overlap at the group level (plants and hummingbirds). Thus, calculating both can aid in understanding the observed changes.

To determine whether the network metrics were the result of chance, we compared each network metric with a null model (a pattern-generating model based on the randomization of ecological data; Fischer & Lindenmayer, 2002; Ulrich, Almeida-Neto & Gotelli, 2009; Ulrich & Gotelli, 2013). Specifically, we assessed the significance of the network metrics, comparing our results to the expectations of the r2dtable null model (*N* = 1,000), which maintains the matrix sum and row/column sums constant (Dormann, Gruber & Fründ, 2008; R Development Core Team, 2017). *P* values are calculated by counting the frequency of matrices in the null model that have higher values than the empirical network. Significant *P* values (*P* ≤ 0.05) indicate the probability that the metrics calculated by the null model are higher than those obtained from the empirical network. Low *P* values indicate that the empirical network metric deviates significantly from the mean metrics obtained by the null model.

To determine differences in the structure of the hummingbird-plant network over time, we compared the observed parameters (connectance, robustness, specialization H_2_′, and niche overlap) to the parameters estimated in randomly generated matrices in R (Patefield, 1981). Specifically, we first obtained the difference between the observed parameter in the 1985–1986 survey and the more recent 2016–2017 survey (e.g., [1985–1986 connectance] − [2016–2017 connectance]). Then, we randomly generated 1,000 matrices using the Patefield algorithm, which generates random matrices with the same matrix sums, including the same row and column sums, from each of the two observed matrices. After this, we calculated the chosen metric for each of the randomly generated matrices and obtained the difference between the 1,000 pairs of matrices (e.g., [connectance in the 1985–1986 random matrix #1] − [connectance in the 2016–2017 random matrix #1]). Finally, we plotted the distribution of these values (1,000 differences) to determine which proportion of these values was smaller than the real (observed) differences. If the observed differences were larger than at least 95% of the differences from the random matrices, we considered them to be significant.

Finally, we calculated nestedness for each network using the NODF parameter (Almeida-Neto et al., 2008) in Aninhado 3.0 (Guimarães & Guimaraes, 2006). This parameter measures the structure of interaction networks, considering a pattern of core generalist species interacting with one another and extreme specialist species interacting only with generalists. Values close to 1 indicate a high degree of nestedness (Bascompte et al., 2003; Guimarães et al., 2007). Since the value of this index is affected by matrix shape and size, we compared this value with the equivalent value of an appropriate null model, which served as a benchmark corresponding with the nested value resulting from a set of probabilistic rules. Accordingly, we were also able to test the statistical significance (Bascompte & Jordano, 2006) of the NODF values (*P* ≤ 0.05) estimated from null model 2 (Bascompte et al., 2003). This model generates random matrices from the original one, and the probability of interaction between any pair of species is considered to be proportional to the total number of interactions (i.e., the degree of interactions). The *P* value was obtained from the proportion of random matrices that had a NODF value equal to or larger than the original matrix.

## Results

**Figure 1:**
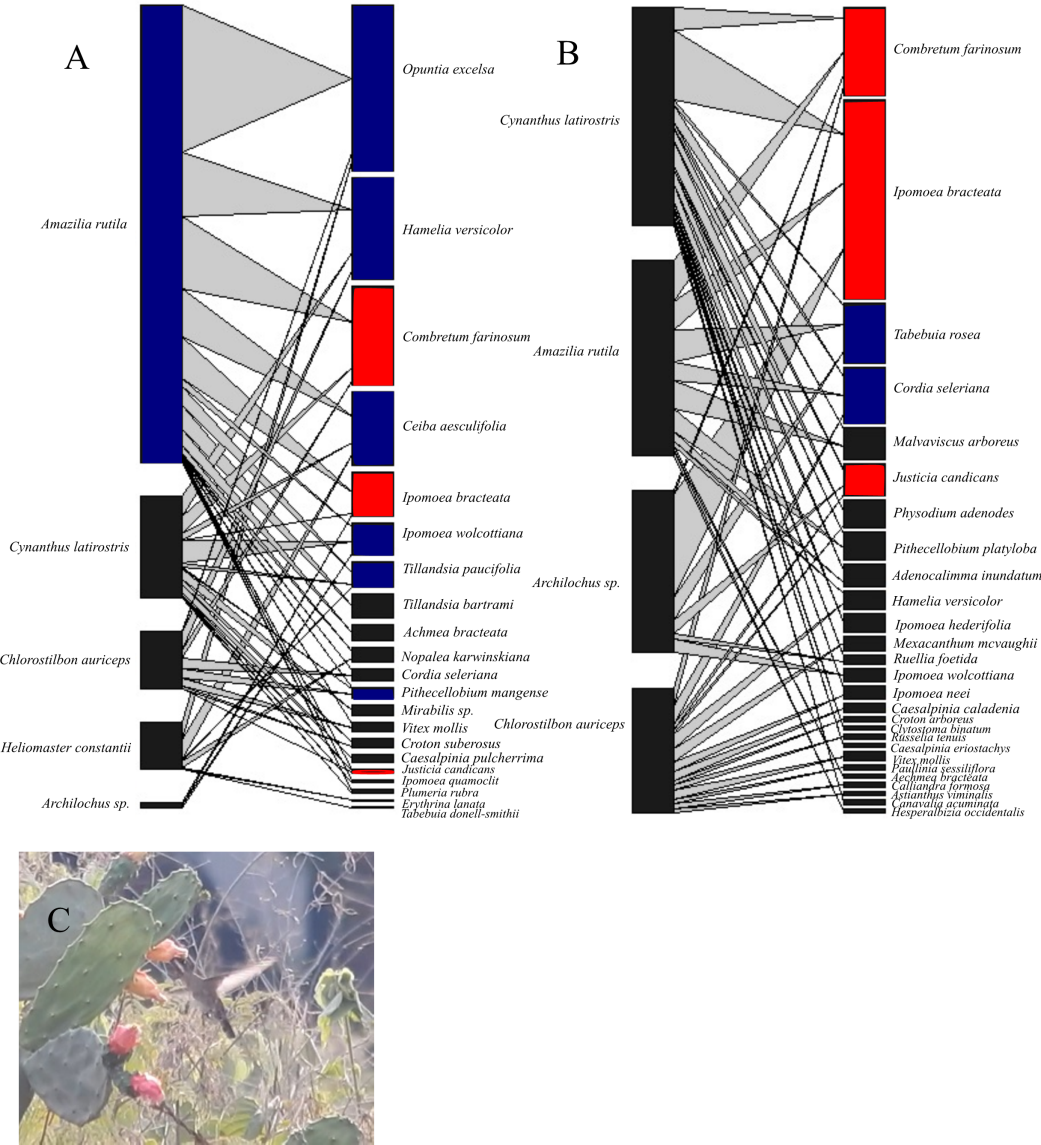
Bipartite hummingbird-plant interaction networks in the Chamela dry forest, Mexico. Nodes on the right of each network represent different plant species and on the left, hummingbird species. Blue nodes represent the core species in a single survey; red nodes represent core species in both surveys. The thickness of each link (gray lines) indicates the frequency of each pairwise interaction (hummingbird flower visits). (A) Network for the 1985–1986 period (before hurricanes). (B) Network for the 2016–2017 period (after hurricanes). (C) *Heliomaster constantii* visiting a *Nopalea karwinskiana* flower. Photograph by Sergio Díaz Infante.

The networks differed in the number of interacting hummingbird and plant species. The 1985–1986 network included 21 plant and 5 hummingbird species, and the 2016–2017 network included 27 plant and 4 hummingbird species. Both networks had 45 interaction links (Fig. 1). A total of 40 plant species were visited by hummingbirds (Table 1), yet only 8 plant species were shared by both periods. In the 2016–2017 network, 13 plant species previously reported in the 1985–1986 network were absent, and 19 new plant species were added. Some plant families that lost species included Cactaceae, Bromeliaceae, and Fabaceae but, in this latter family, several new species were also added. Conversely, of the 5 hummingbird species registered in the 1985–1986 network, only *Heliomaster constantii* was absent in the 2016–2017 network. Because of the difficulty of correctly distinguishing *Archilochus colubris* and *A. alexandri* females and juveniles under field conditions, both species were conjointly considered in the analyses.

We observed several changes in the core and periphery species of both networks (Fig. 1). Of the hummingbird species in the 1985–1986 network, *Amazilia rutila* (*Gc* = 1.25) was the only core species, whereas in the 2016–2017 network, none of the hummingbirds were considered to be core species, even though *Cynanthus latirostris* (*Gc* = 0.91) obtained a *Gc* value near one. Of the plant species in the 1985–1986 network, 9 out of 21 species (42.8%) belonged to the generalist core, whereas in the 2016–2017 network, only 5 out of 27 species (18.5%) belonged to this group. Of the original plant species core, only *C. farinosum*, *I. bracteata*, and *J. candicans* maintained this status in the 2016–2017 network.

Most network parameters were similar in both sampling periods (Table 2). However, robustness in plant species differed (0.88 and 0.93, *P* = 0.004), demonstrating that plants became 5.6% more robust to extinctions in the second sampling period. Niche overlap in hummingbirds also increased significantly (51.8%) from the first period to the second (0.27 and 0.41, *P* = 0.03), but we did not observe a difference in plants (0.41 and 0.36, *P* = 1.0).

**Table 1:** Plant species visited by hummingbirds (x) in the 1985–1986 and 2016–2017 sampling periods. Plant species in bold indicate those recorded in both periods.

Family	*Plant species*	1985–1986	2016–2017		Growth form		
Acanthaceae	***Justicia candicans***	x	x		Shrub		
	*Mexacanthus mcvaughii*		x		Shrub		
	*Ruellia foetida*		x		Shrub		
Apocynaceae	*Plumeria rubra*	x			Tree		
Bignonaceae	*Astianthus viminalis*		x		Tree		
	*Adenocalymma inundatum*		x		Vine		
	*Clytostoma binatum*		x		Vine		
	*Tabebuia donnell-smithii*	x			Tree		
	*Tabebuia rosea*		x		Tree		
Boraginacea	***Cordia seleriana***	x	x		Tree		
Bromeliaceae	***Aechmea bracteata***	x	x		Epiphyte		
	*Tillandsia bartramii*	x			Epiphyte		
	*Tillandsia paucifolia*	x			Epiphyte		
Cactaceae	*Nopalea karwinskiana*	x			Cactus		
	*Opuntia excelsa*	x			Cactus		
Combretaceae	***Combretum farinosum***	x	x		Vine		
Convolvulaceae	***Ipomoea bracteata***	x	x		Vine		
	*Ipomoea hederifolia*		x		Vine		
	*Ipomoea neei*		x		Vine		
	*Ipomoea quamoclit*	x			Vine		
	***Ipomoea wolcottiana***	x	x		Tree		
Euphorbiaceae	*Croton arboreus*		x		Tree		
	*Croton suberosus*	x			Shrub		
Fabaceae	*Pithecellobium platyloba*		x		Vine		
	*Caesalpinia caladenia*		x		Tree		
	*Caesalpinia eriostachys*		x		Tree		
	*Caesalpinia pulcherrima*	x			Shrub		
	*Canavalia acuminata*		x		Vine		
	*Erythrina lanata*	x			Tree		
	*Hesperalbiziaoccidentalis*		x		Tree		
	*Calliandra formosa*		x		Shrub		
	*Pithecellobium mangense*	x			Tree		
Malvaceae	*Ceiba aesculifolia*	x			Tree		
	*Malvaviscus arboreus*		x		Shrub		
Nyctaginaceae	*Mirabilis sp.*	x			Herb		
Rubiaceae	***Hamelia versicolor***	x	x		Shrub		
Sapindaceae	*Paullinia sessiliflora*		x		Vine		
Scrophullariaceae	*Russelia tenuis*		x		Herb		
Sterculiaceae	*Physodium adenodes*		x		Shrub		
Verbenaceae	***Vitex mollis***	x	x		Tree		

## Discussion

We found that natural disturbance events such as hurricanes may have impacted some of the structural parameters of the studied hummingbird-plant network in Chamela, Mexico. Plant robustness and hummingbird niche overlap increased after the disturbance events, and the generalist species core of the network was reduced in number. However, overall, the number of species in the interaction network after the disturbance events was larger than in the prior survey because of the inclusion of new plant species, even though many of the plant species present in the first survey were lost. Notably, four out of five hummingbird species were retained in the second survey, with the exception of *Heliomaster constantii*.

Temporal changes in the number and identity of species forming interaction networks have been previously reported. For example, a three-year study in California (United States) registered changes in species interactions, although few differences were noted in the number of plants and pollinators (Alarcón, Waser & Ollerton, 2008). Dupont et al. (2009) studied pollination networks across different latitudes and found that species identity and interactions changed from one year to the next, although the number of species and interactions remained constant. Petanidou et al. (2008) reported that most species and interactions changed over a four-year period near Athens (Greece): Only 53% of plant species, 21% pollinator species, and 4.9% of interactions remained constant. Díaz-Castelazo et al. (2010) showed that, over a period of 10 years, ant-plant networks in Veracruz (Mexico) differed in the number of species and interactions, suggesting a high species turnover. These findings suggest that the actual range and strength of interactions in the plant and pollinator community vary over time and are unlikely to be detected over short periods of time (Alarcón, Waser & Ollerton, 2008). Long-term studies on plant–pollinator networks are needed, especially after disturbance events such as droughts and hurricanes, as the composition of interacting species can change following these events. Additionally, the modification of plant abundance, flowering, or pollinator phenology as a result of climatic changes can affect the plant–pollinator network (Burkle & Alarcón, 2011).

**Table 2:** Parameters of the plant-hummingbird interaction network in the 1985–1986 and 2016–2017 sampling periods. Numbers in bold indicate significant values (*P* ≤ 0.05). Asterisks (*) indicate significant differences (*P* ≤ 0.05) in a specific attribute between periods.

Period	1985–1986	*P*	2016–2017	*P*
Plant species	21		27	
Hummingbird species	5		4	
Connectance	**0.43**	<0.05	**0.42**	<0.05
Robustness (hummingbirds)	0.68	0.42	0.62	0.29
Robustness (plants)	0.87^*^	0.42	0.93^*^	0.29
Specialization H_2_’	**0.41**	0.05	0.05	0.43
Niche overlap (hummingbirds)	0.27^*^	0.09	0.41^*^	0.41
Niche overlap (plants)	0.42	0.09	0.36	0.41
Nestedness (NODF)	48.61	0.48	41.88	0.66

Two epiphytic plant species were lost in the 2016–2017 interaction network including *T. paucifolia*, a generalist core species in the 1985–1986 network. Extreme events can cause the direct mortality of epiphytic plants or damage their host trees (Robertson & Platt, 2001). In Chamela’s dry forest, droughts are part of the intra- and interannual dynamics of the region (Martijena & Bullock, 1994; Maass et al., 2017); thus, the inhabiting species are likely well adapted to these events. However, the abundance of epiphytic species was impacted, for example, after Hurricanes Andrew, Hugo, and Wilma in the Caribbean (Migenis & Ackerman, 1993; Loope et al., 1994; Goode & Allen, 2008). As a result of climate change and the corresponding predicted increase in hurricane incidence, epiphytic plants may disproportionally suffer compared to other understory plant species.

Cacti adapted to drought conditions such as *Nopalea karwinskiana* and *Opuntia excelsa* also disappeared from the 2016–2017 network. The first cactus was restricted to rocky creek edges yet, after unusually heavy rains in 1998, it completely disappeared (MC Arizmendi, pers. comm., 1998). It was a periphery species but was important for *H. constantii* (Arizmendi & Ornelas, 1990), which was not registered in the second survey. Many *O. excelsa* individuals (a generalist core plant species, particularly important for *H. constantii* and *A. rutila* hummingbirds) fell or lost their terminal cladodes (those that produce flowers) due to wind force and felled trees after Hurricane Patricia (S Díaz Infante, pers. comm., 2016), as reported for other similar events (Frangi & Lugo, 1991). The effects after Hurricane Jova were probably similar but likely occurred at a lower intensity due to the lower wind force of this hurricane.

Most of the rest of missing plants in the 2016–2017 network had few interactions and were periphery species loosely connected to hummingbirds, so their probability of interaction with hummingbirds was already low (Borgatti & Everett, 2000). Notably, blooming was not observed in tall trees such as *Tabebuia donell-smithii* and *P. mangense* during the 2016–2017 survey. These species are more susceptible to damage and mortality as a result of strong winds during hurricanes (Wurman & Winslow, 1998; Segura et al., 2002; Vandecar et al., 2011). Trees such as *Erythrina lanata* and *Ceiba aesculifolia*, located on slopes and hills, did not bloom either after Hurricane Patricia, as most of their treetops were removed after the hurricane. This probably also occurred with Hurricane Jova. Parker et al. (2017) found that both hurricanes altered the forest structure in Chamela, but Hurricane Patricia had a larger and more devastating effect on fruit and flower abundance and phenology than Hurricane Jova (Renton et al., 2017).

Most of the new plant species added to the network are mainly pollinated by insects. They had few interactions with hummingbirds, and none belonged to the generalist core. Thus, the probability of detecting their interactions before was also low (Van der Pijl, 1961; Tripp & Manos, 2008). Moreover, most new species were vines or shrubs (8 spp.), which likely benefitted from the newly opened gaps after Hurricane Patricia. For example, even though the upper strata (4 to 11 m) lost up to 83% ± 16% of canopy material, the lower strata (1 to 3 m) increased its cover by 61% ± 68% after the hurricane, mainly due to the growth of vines and shoots (Parker et al., 2017). One unique case is *Tabebuia rosea*, a tree species damaged by wind but included as a core species in the second survey; most registered individuals were planted after the first survey (JM Verdusco, pers. comm., 1987).

Overall, our data concur with reports in other study sites affected by hurricanes. For example, the number of fruits and flowers on woody plants was abnormally low several months after Hurricane Hugo impacted the Virgin Islands (Askins & Ewert, 1991); Waide1991). Likewise, vines, branches, and invasive herbs formed an almost impenetrable mass in the understory of a tropical forest in Quintana Roo, Mexico, several months after the arrival of Hurricane Gilbert (Lynch, 1991). After Hurricane Hugo in Jamaica, the lower strata of foliage recovered faster than the upper strata because of the more rapid growth of herbaceous plants and the sprouting of trees and shrubs. Even after 17 months, treetops had not recovered their original density due to fallen trees and broken trunks and branches (Wunderle Jr, 1995). Similarly, after Hurricane Gilbert in Puerto Rico, understory growth compensated for the loss of treetops (Wunderle Jr, Lodge & Waide, 1992). However, despite the severe damage to vegetation, the species composition of the studied plant communities was speculated to remain unaltered after Hurricanes Hugo and Gilbert (Brokaw & Walker, 1991). In our study, an important fraction of the species absent in the 2016–2017 network were either susceptible to hurricane damage or had low interaction probabilities. Of the newly added species, many had few interactions or were herbs, vines, or shrubs, which appear to have benefitted from the passage of the hurricanes. According to Olesen et al. (2008), the new species added in a network are usually specialized or often rare.

As with plants, the hummingbird species that were originally more connected (*A. rutila*, *C. latirostris*, and *C. auriceps*) were maintained in the network, whereas the species with fewer interactions (*H. constantii* ) and those associated with disturbance-vulnerable plant species such as *O. excelsa*, *C. aesculifolia*, and *N. karwinskiana* were absent. *Amazilia rutila*, the only core species in the first network, was closely associated with *O. excelsa*, a plant affected by hurricane winds. Previous studies have shown that frugivorous and nectarivorous birds are more susceptible to a decline in food resources than other birds, as occurred on the Virgin Islands with the hummingbird *Orthorhynchus cristatus* after Hurricane Hugo (Askins & Ewert, 1991) and in the Yucatán Peninsula in Mexico with nectarivorous birds in general after Hurricane Gilbert (Lynch, 1991). Also, rare hummingbird species are more susceptible to disappearance from an affected area, as occurred with the Vervain Hummingbird (*Mellisuga minima*) in Jamaica after Hurricane Gilbert. However, even abundant hummingbirds, such as the Streamertail Hummingbird (*Trochilus polytmus*), may suffer considerable mortality from exposure to hurricanes or may wander to nearby less damaged areas(Wunderle Jr, Lodge & Waide, 1992), especially considering that tropical nectarivorous birds have a wide range and often migrate seasonally and altitudinally in some tropical regions (e.g., Stiles, 1983).

On the other hand, the two *Archilochus* species seemed to benefit from hurricane-borne disturbance in our study. These generalist winter migrant hummingbirds are commonly associated with disturbed areas (Finch, 1991; Udvardy & Farrand, 1994; Arizmendi & Berlanga, 2014) and mainly associated with the vine *Ipomoea bracteata*. Previously, according to Lynch (1991), overwintering Nearctic migrants appeared to be more resilient than year-round residents to hurricane effects. The latter author found that these migrants, who are generally associated with edges, dense scrub, and open zones, invaded a forest damaged by Hurricane Gilbert in Quintana Roo, Mexico. Thus, it seems that migratory generalist species are favored by disturbance events and the growth and blooming of vines and shrubs.

With respect to the parameters of the hummingbird-plant interaction network, only plant robustness and hummingbird niche overlap presented significant differences after 30 years. Several studies describing temporal patterns in pollination networks revealed similarities (Burkle & Alarcón, 2011). For example, most studied pollination networks have been shown to be highly nested over time (Alarcón, Waser & Ollerton, 2008; Petanidou et al., 2008; Dupont et al., 2009; Díaz-Castelazo et al., 2010). Connectance values also tend to be conserved over time (Alarcón, Waser & Ollerton, 2008; Olesen et al., 2008; Petanidou et al., 2008; Dupont et al., 2009; Díaz-Castelazo et al., 2010). As sampling time increases, specialization tends to diminish (Petanidou et al., 2008) because pollinators may use less preferred floral resources when their favorite plant species are scarce or absent (Burkle & Alarcón, 2011). However, interaction network characteristics appear to remain constant within a framework of annual variation in species identity (Petanidou et al., 2008). In our study, the generalist core of nine plants in 1985–1986 was reduced to five in 2016–2017; only three of the original species were conserved. Also, the only former core hummingbird species (*A. rutila*) lost this status in 2016–2017, probably due to the disappearance of two core plant species (*O. excelsa* and *C. aesculifolia*) that served as resources for this hummingbird, and *H. constantii* was also lost after the hurricanes. In southern California (United States), the identity of core species also changed in plant–pollinator networks studied over three years (Alarcón, Waser & Ollerton, 2008), indicating that specialists interact with different groups of generalists in different years given that even generalist species are subject to fluctuations in population size.

The fact that mutualistic networks form well-defined and predictable patterns of interdependence supports a community-wide approach for species conservation (Bascompte & Jordano, 2007). Network analyses can contribute to conservation strategies at the community level and can highlight important species for the network that should be conserved and protected (Lara-Rodríguez et al., 2012). In addition, the most relevant species in terms of abundance and interaction frequencies can provide insights into the ecological and evolutionary history of interaction networks (Bascompte & Jordano, 2007). Theoretical evidence concludes that heterogeneous, nested, mutualistic networks confer network robustness to species loss and habitat fragmentation (Memmott, Waser & Price, 2004; Fortuna & Bascompte, 2006; Burgos et al., 2007).

However, overall robustness to random species extinctions may be partly explained by the role of a few highly connected species in a network’s core with which specialists interact (Bascompte, 2009). Thus, the robustness of an entire network depends on these species. In Chamela, *I*. *bracteata*, *C. farinosum*, and *J. candicans* remained as core species overtime, even after extreme events. Therefore, these species significantly contributed to the connectivity and resilience of the studied plant-hummingbird network (Sánchez-Galván, Díaz-Castelazo & Rico-Gray, 2012). Moreover, an increase in hummingbirds’ niche overlap suggests that generalist pollinators can expand their diet (e.g., *Archilochus spp*. and *C. auriceps*) when resources are scarce (Waser, 1986), thereby increasing plant robustness. In conclusion, it appears that, despite the disturbances caused by hurricanes, most plant species survive (Burgos et al., 2007), especially if the remaining pollinators become more generalist.

We also considered other factors that could have caused the observed changes in the interaction networks, even though we did not register plant-hummingbird interactions immediately before the hurricanes. However, to the best of our knowledge, no other natural or human-induced disturbances could be directly related to the observed changes in this well-studied site (besides the ones already discussed). On the other hand, we have previous evidence supporting that hurricanes shape forest structure, influence their species composition and diversity, and regulate their functioning (Lugo, 2008).

As stated before, some severe droughts were recorded at the study site, but plants and animals native to the area are adapted to a highly unpredictable and strongly seasonal rainfall pattern (Maass et al., 2017). Drought can affect plants’ flowering, but its effects in dry forests are usually restricted to the seasons/years when it occurs and the following year/season. Thus, if plants are able to resist drought, there is usually not a lasting effect on flowering. Furthermore, in the last 33 years, there have not been any reported vegetation changes in the area due to drought. Thus, we can assume that the reason that many plants or trees did not bloom during the second survey was hurricane damage rather than drought, especially considering that these effects are not normal in the study site. Finally, we were able to directly observe damage to the vegetation cover that was also reported in the literature (Maass et al., 2017; Jimenez-Rodríguez et al., in press; Parker et al., 2017).

Even so, distinguishing the effects of a large, infrequent event from the background variation of a seasonal ecosystem is challenging (Parker et al., 2017). Thus, we recognize that only longer-term studies would evidence network fluctuations due to normal inter-annual variation in the abundance and phenology of plant and hummingbird populations. Also, a recovery of vegetation and decrease in new opportunistic species would confirm that natural disturbances had been influencing the interaction network.

However, some external factors not considered herein may also contribute to the observed interactions (e.g., external effects on migratory hummingbirds). Either way, if plant–pollinator interactions are dynamic and opportunistic, this should increase the resistance of pollination networks to species loss and phenological changes (Alarcón, Waser & Ollerton, 2008). Thus, even in the face of extreme events, these networks may persist.

## Conclusions

The number and identity of interacting species in the hummingbird-plant network of a dry forest in Chamela, Mexico, primarily plant species, changed over a 30-year period. We mostly observed changes in the less connected species and those more prone to be affected (e.g., epiphytes, cacti, and large trees) or benefitted (e.g., herbs, vines, and shrubs) by hurricanes. In the case of hummingbirds, all but one (*Heliomaster constantii*) remained in the network over time. Overall, most network structural parameters remained unchanged, with the exception of plant robustness and hummingbird niche overlap, which both increased. Also, extreme events appear to have impacted the generalist core of species, resulting in the loss of several species. Finally, the most important plant species for conserving the plant-hummingbird interaction network in the Chamela-Cuixmala Biosphere Reserve are *Ipomoea bracteata*, *Combretum farinosum*, and *Justicia candicans*. The temporal perspective of this study provides unique insights into the conservation of plant-hummingbird networks across time and after extreme natural events.

##  Supplemental Information

10.7717/peerj.8338/supp-1 Supplemental Information 1 Hummingbird-plant interactions recorded from 1985-1986 in Chamela, Mexico Column heads represent hummingbird species. Raw heads represent plant species. Numbers represent interactions.

10.7717/peerj.8338/supp-2 Supplemental Information 2 Hummingbird-plant interactions recorded from 2016-2017 in Chamela, Mexico Column heads represent hummingbird species. Raw heads represent plant species. Numbers represent interactions.

10.7717/peerj.8338/supp-3 Supplemental Information 3 R code for interaction network analyses R code for bipartite analyses

## Additional Information and Declarations

### Data Availability

The following information was supplied regarding data availability: The matrices with raw interactions from both surveys and the R code for the analyses are available in the Supplementary Files.
